# Concurrent use of anlotinib overcomes acquired resistance to EGFR‐TKI in patients with advanced *EGFR*‐mutant non‐small cell lung cancer

**DOI:** 10.1111/1759-7714.14141

**Published:** 2021-09-12

**Authors:** Chen Zhang, Honggang Cao, Yanan Cui, Shidai Jin, Wen Gao, Chenjun Huang, Renhua Guo

**Affiliations:** ^1^ Department of Medical Oncology the First Affiliated Hospital of Nanjing Medical University Nanjing China; ^2^ Department of Medical Oncology the Third People's Hospital of Yancheng Yancheng China; ^3^ Department of Cardiothoracic Surgery, The First Affiliated Hospital Nanjing Medical University Nanjing China

**Keywords:** acquired resistance, anlotinib, EGFR‐TKI, non‐small cell lung cancer, VEGFR

## Abstract

**Background:**

Acquired resistance development is a major challenge in the epidermal growth factor receptor‐tyrosine kinase inhibitor (EGFR–TKI) treatment of non–small cell lung cancer (NSCLC). Here, we investigated the potential effects of the concurrent use of anlotinib and EGFR‐TKI to overcome acquired resistance.

**Methods:**

We conducted a preclinical study to evaluate the antitumor effects of gefitinib + anlotinib in gefitinib‐resistant lung adenocarcinoma models in vitro and in vivo. We then investigated the treatment effect of EGFR–TKI + anlotinib therapy in 24 advanced *EGFR*‐mutant NSCLC patients after EGFR‐TKI acquired resistance between January 2018 and August 2020.

**Results:**

Anlotinib reversed gefitinib resistance in gefitinib‐resistant lung adenocarcinoma models by enhancing the antiproliferative and proapoptotic effects of gefitinib. The gefitinib + anlotinib treatment exerted a synergistic antitumor effect by downregulating the activation of VEGFR2 and downstream effectors, Akt and ERK. The EGFR–TKI + anlotinib therapy exhibited an objective response rate of 20.8% and a disease control rate of 95.8%. Median progression‐free survival (PFS) was 11.53 ± 2.41 months, but median overall survival was not reached. The median PFS was longer in patients experiencing gradual progression (13.30 ± 1.69 months) than in patients with dramatic progression (6.80 ± 1.75 months, *p* = 0.017). One grade 3 adverse event was noted (diarrhea, *n* = 2, 8.3%), and grade 4 or 5 adverse events were absent.

**Conclusions:**

EGFR–TKI combined with anlotinib demonstrated powerful antitumor activity in vitro and in vivo. Concurrent use of anlotinib overcomes acquired resistance to EGFR‐TKI in advanced *EGFR*‐mutant NSCLC patients.

## Graphical abstract

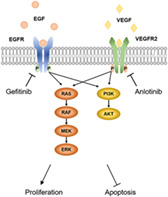

## INTRODUCTION

Lung cancer is one of the most fatal cancers worldwide, and non–small cell lung cancer (NSCLC) is the most common histological type accounting for up to 85% of lung cancer cases.^1^, ^2^ The investigation of lung cancer pathogenesis and identification of various therapeutic molecular targets has increased the significance of targeted therapy in NSCLC. The most widely used targeted agents, epidermal growth factor receptor–tyrosine kinase inhibitors (EGFR–TKIs), have been determined to significantly prolong survival in *EGFR*‐mutant patients with NSCLC.^3^, ^4^ However, the clinical benefits of EGFR–TKI therapies are challenged by acquired resistance. Several mechanisms leading to acquired resistance have been identified, including secondary *EGFR* T790M mutation, bypass track signaling pathways such as MET amplification and HER2 amplification, phenotypic change to small‐cell lung cancer, epithelial‐to‐mesenchymal transformation, and so on.^5^ Several approaches have been developed to overcome acquired resistance, such as adopting third‐generation EGFR‐TKIs^6^ and combining them with bypass signaling pathway inhibitors.^7^ Switching platinum‐based chemotherapy is recommended for patients who develop resistance with an unknown mechanism, because no targeted treatment options are currently available.^8^

Vascular endothelial growth factor receptor (VEGFR) is another significant target for the treatment of NSCLC. Molecular research shows that VEGFR and EGFR share common signaling pathways such as PI3K/Akt and mitogen‐activated protein kinase signaling pathways. EGFR inhibition can downregulate VEGF expression by hypoxia‐inducible factor (HIF)‐1‐independent and HIF‐1‐dependent mechanisms.^9^ Locally secreted VEGF promotes angiogenesis in the tumor microenvironment and is involved in EGFR–TKI resistance.^10^ Based on these findings, we speculated that the dual targeting of EGFR and VEGFR may be theoretically effective in patients with acquired EGFR–TKI resistance. Numerous recent clinical trials support our hypothesis. Afatinib plus bevacizumab (a potent monoclonal antibody targeting VEGF) have reported positive treatment effect in patients with NSCLC after acquired resistance to EGFR–TKI.^11^ Apatinib (a small‐molecule TKI that blocks VEGFR/PDGFR/c‐Kit/RET/Src) has also been reported to enhance the antitumor activity of EGFR–TKI in NSCLC with acquired resistance.^12^

Anlotinib is a novel oral multitargeted small‐molecular TKI approved for the treatment of third‐line advanced NSCLC.^13^ Anlotinib mainly exerts its antiangiogenic and broad‐spectrum antitumor effects by the highly potent and specific suppression of VEGFR2.^14^ The ALTER 0303 clinical trial demonstrated favorable outcomes in patients with advanced NSCLC.^15^ These facts motivated us to explore a new therapeutic option to overcome acquired resistance by combining EGFR‐TKI and anlotinib.

We carried out a preclinical study to demonstrate the synergistic effect of gefitinib combined with anlotinib in resistant lung adenocarcinoma models. We then investigated the treatment effect of EGFR–TKI + anlotinib therapy in patients with advanced *EGFR*‐mutant NSCLC after acquired resistance.

## METHODS

### Cell lines and reagents

Human NSCLC cells, PC9, were purchased from the Institute of Biochemistry and Cell Biology at the Chinese Academy of Sciences (Shanghai, China). The gefitinib‐resistant cells, PC9/GR, were provided by Shanghai Pulmonary Hospital. All the cells were cultured in RPMI 1640 media supplemented with 10% fetal bovine serum and antibiotics (penicillin 100 U/ml and streptomycin 100 mg/ml).

Gefitinib (CAS no. 184475‐35‐2) and anlotinib (CAS no. 1360460‐82‐7) were purchased from Selleck chemicals LLC. All chemicals were dissolved in dimethyl sulfoxide (DMSO) for in vitro experiments and in sodium carboxyl methyl cellulose (CMC‐Na) (1%) for in vivo experiments. Antibodies against EGFR, phosphorylated EGFR (p‐EGFR), VEGFR2, phosphorylated VEGFR2 (p‐VEGFR2), ERK1/2, phosphorylated ERK1/2 (p‐ERK1/2), Akt, phosphorylated Akt (p‐Akt), and cleaved‐caspase 3 were obtained from Cell Signaling Technology.

### Cell proliferation assays

#### Cell viability assay

Cell viability was measured by cell counting kit‐8 (CCK8: Selleck) assay. PC9 and PC9/GR cells were plated in 96‐well plates at a density of 3 × 10^3^/well and incubated overnight. The cells were subsequently exposed to increasing concentrations of gefitinib (0.01, 0.1, 1.0, 2.0, 5.0, and 10.0 μM), anlotinib (1.0, 2.0, 4.0, 8.0, 16.0, and 32.0 μM), or a gefitinib–anlotinib combination (gefitinib: 0.01, 0.1, 1.0, 2.0, 5.0, and 10.0 μM; with anlotinib 2.0 μM) for 72 h. Then, 10 μl of CCK8 was added into each well and incubated for 1 h. The absorbance (optical density, OD) at 450 nm was detected using a microplate reader (Tecan). Cell viability was calculated as: cell viability = (OD_sample_ − OD_blank_)/(OD_control_ − OD_blank_) × 100%. The values of the half maximal inhibitory concentration (IC50) were calculated by GraphPad Prism 8.3.0 (GraphPad Software). Experiments were repeated in triplicate, and an average was obtained. The combination index for the combination of gefitinib and anlotinib was calculated using CompuSyn software.

#### 5‐ethynyl‐20‐deoxyuridine cell proliferation assay

The PC9/GR cells were cultured in 12‐well plates at 1 × 10^5^ cells/well overnight. Subsequently, the cells were exposed to DMSO, 0.1 μM gefitinib, 2 μM anlotinib, or a combination of 0.1 μM gefitinib and 2 μM anlotinib for 48 h. The ethynyl‐20‐deoxyuridine (EdU) incorporation assay was performed using an EdU assay kit (C0071S) according to the manufacturer's instructions. Then, 10 μM EdU was added to each well and 2 h allowed for incorporation. After treatment with 4% paraformaldehyde and 0.3% Triton X‐100, the cells were stained with anti‐EdU click additive solution. Hoechst 33342 was used to label the cell nuclei. The percentage of EdU‐positive cells was calculated after fluorescence microscopy analysis. Three fields of view were randomly assessed for each treatment group.

#### Colony forming assay

PC9/GR cells were seeded in 6‐well plates at 500 cells/well overnight. They were then treated with DMSO, 0.1 μM gefitinib, 2 μM anlotinib, or a combination of 0.1 μM gefitinib and 2 μM anlotinib for 24 h. The medium was replaced every 3 days. After 10 days, the colonies were fixed with methanol and stained with a 0.1% crystal violet (Sigma). Total colonies with diameter > 0.5 mm were counted. Experiments were repeated in triplicate, and an average was obtained.

### Cell apoptosis detection

PC9/GR cells were seeded overnight in 6‐well plates at 1 × 10^5^ cells/well. They were then treated with DMSO, 0.1 μM gefitinib, 2 μM anlotinib, or a combination of 0.1 μM gefitinib and 2 μM anlotinib. Cell apoptosis detection was performed using Annexin V‐FITC/propidium iodide (PI) Apoptosis Detection Kit (40302, Yeasen) according to the manufacturer's instructions. The treated cells were briefly harvested and washed with phosphate‐buffered saline, followed by suspension in 100 μl of Annexin V binding buffer. Then, 5 μl of Annexin V‐FITC and 10 μl of PI were added for incubation in the dark. Samples were then analyzed using a flow cytometer (FACScan, BD Biosciences). Experiments were performed in triplicate, and an average was obtained.

### Protein expression by western blotting

The total cellular protein lysates of PC9 and PC9/GR cells were separated on 10% sodium dodecyl sulfate polyacrylamide gel electrophoresis and transferred to polyvinylidene fluoride membranes (Millipore). The membranes were incubated with specific antibodies against EGFR, p‐EGFR, VEGFR2, p‐VEGFR2, ERK1/2, p‐ERK1/2, Akt, p‐Akt, and cleaved‐caspase 3 overnight at 4°C. Glyceraldehyde 3‐phosphate dehydrogenase was used as an internal control for EGFR, p‐EGFR, VEGFR2, p‐VEGFR2, ERK1/2, p‐ERK1/2, Akt, and p‐Akt, whereas β‐actin was used as an internal control for cleaved‐caspase 3. The immunoreactive bands were visualized with enhanced chemiluminescence (ECL) using an ECL detection system. The band densities were quantified using ImageJ (NIH). Experiments were performed in triplicate, and an average was obtained.

### Tumor formation assay in nude mouse model

Five‐week‐old male athymic BALB/c mice were maintained under specific pathogen‐free conditions and manipulated in accordance with an Institutional Animal Care and Use Committee (IACUC)–approved protocol (IACUC protocol number: 2102023). PC9/GR cells at a concentration of 2 × 10^7^/ml were injected into either side of the posterior flank of the mice in a 100 μl volume. The tumor volumes (0.5 × length × width^2^) were measured every 3–4 days. When solid tumor reached an average volume of 50 mm^3^, mice were treated daily by oral gavage as follows: (a) saline for 21 days; (b) gefitinib (25 mg/kg) alone for 21 days; (c) anlotinib (6 mg/kg) alone for first 14 days; (d) gefitinib (25 mg/kg) for 21 days combined with anlotinib (6 mg/kg) for first 14 days. After 21 days, the tumors were resected from all of the mice. Tumor weights were measured, and tumour tissues were used for hematoxylin‐eosin (HE) and immunohistochemical (IHC) staining.

### Clinical data collection

#### Study population and treatment

This retrospective study was conducted in the department of Medical Oncology at the First Affiliated Hospital of Nanjing Medical University between January 2018 and August 2020. Eligible patients were cytologically‐ or histologically‐confirmed with advanced *EGFR*‐mutant NSCLC and had exhibited acquired resistance to first‐ or second‐generation EGFR–TKI, according to the criteria by Jackman et al.,^16^ with no targetable resistance mechanism identified by rebiopsy after acquired resistance. All patients were fully informed and signed an informed consent form before continuation of EGFR‐TKI treatment and concurrent use of anlotinib (Chia Tai Tianqing Pharmaceutical Group Co.). EGFR–TKI was continued with original dose and schedule, and anlotinib (one cycle of 10 mg per os daily for 14 days, discontinued for 7 days, and repeated every 21 days) was added until disease progression or intolerable toxicity. The dosage adjustments (increase or decrease) were decided by the attending physician according to clinical response and adverse events. The medical records of patients were collected to study related characteristics such as age, sex, histological type, *EGFR* mutation type, and prior EGFR–TKI treatment. This study was approved by the Ethics Committee of the First Affiliated Hospital of Nanjing Medical University.

#### Assessment of treatment effect and adverse events

Tumor assessment was performed using radiographic data according to the Response Evaluation Criteria in Solid Tumor (RECIST), version 1.1. The modes of EGFR–TKI treatment failure were divided into dramatic progression (disease control lasting ≥3 months; rapid increment of tumor burden compared with the previous assessment; symptom scored 2), gradual progression (disease control lasting ≥6 months; minor increment of tumor burden compared with the previous assessment; symptom scored ≤1), and local progression (disease control lasting ≥3 months; solitary extracranial or intracranial progression; symptom scored ≤1) according to the criteria of Yang et al.^17^ Progression‐free survival (PFS) was defined as the time from the initiation of combination therapy of EGFR‐TKI and anlotinib until the first observed progression or death. Overall survival (OS) was defined as the time from the initiation of combination therapy until death. The adverse events were assessed according to the Common Terminology Criteria for Adverse Events (CTCAE, version 5.0).

### Statistical analysis

Statistical analysis was performed using SPSS version 23.0 (IBM) and GraphPad Prism 8.3.0 (GraphPad Software). Continuous variables are presented as means ± standard deviations, or as median along with the minimum–maximum range, if not normally distributed. Categorical variables are described as n (%). Student's *t* test was used for continuous variables. PFS and OS were estimated using the Kaplan–Meier method. Subgroup analyses were performed by *EGFR* mutation type and EGFR–TKI failure mode. The PFS between groups was compared using the log‐rank test. A *p* value of <0.05 was considered statistically significant.

## RESULTS

### Resensitization of PC9/GR cells to gefitinib by anlotinib

**FIGURE 1:**
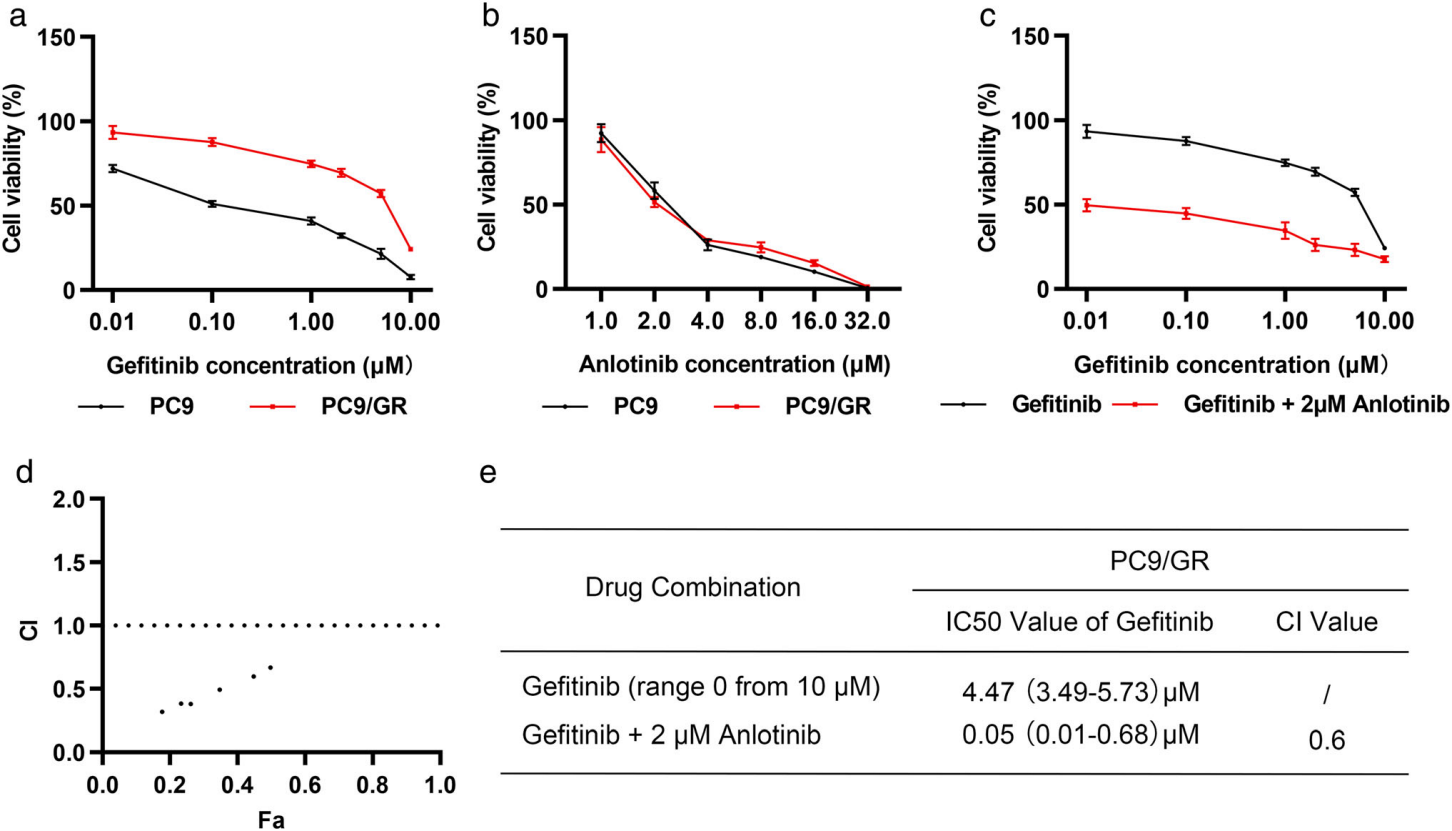
Anlotinib resensitized gefitinib‐resistant PC9/GR cells to gefitinib. (a–c) Parental lung adenocarcinoma PC9 cells and gefitinib‐resistant PC9/GR cells were treated with indicated concentrations of gefitinib (a), anlotinib (b), or gefitinib plus anlotinib (c) for 72 h. Cell viability was measured with a CCK8 assay. (d) The combination index (CI) of gefitinib plus anlotinib was calculated using CompuSyn software. (e) Half maximal inhibitory concentration (IC50) and CI values of gefitinib alone or combined with anlotinib in PC9/GR cells

To demonstrate the resistance of PC9/GR cells to gefitinib, we treated PC9 and PC9/GR cells with gefitinib for 72 h and detected cell viability through the CCK8 assay. IC50 values in PC9/GR cells (IC50: 4.47 μM, 95% confidence interval [CI]: 3.49–5.73 μM) were approximately 9‐fold higher than those in the gefitinib‐sensitive cell line PC9 (IC50: 0.49 μM, 95% CI: 0.09–1.19 μM) (Figure 1a).

As a multitargeted small‐molecular TKI, anlotinib induced similar inhibitory effects on PC9 and PC9/GR cell viability (Figure 1b; IC50: 2.53 μM, 95% CI: 1.88–3.39 μM in PC9 vs. IC50: 2.51 μM, 95% CI: 1.93–3.26 μM in PC9/GR). Moderate inhibitory effects were exhibited in both cell lines (about 41.6% on PC9 cells and 48.5% on PC9/GR cells) by 2 μM anlotinib. Therefore, it was selected for the combination experiments. Gefitinib significantly inhibited cell growth in PC9/GR cells when used in combination with 2 μM anlotinib (Figure 1c; IC50 of gefitinib: 0.05 μM, 95% CI: 0.01–0.68 μM).

The combination index was calculated using the Chou–Talalay method to determine whether the combined effect was synergistic. The calculated combination indices ranged from 0.3 to 0.7 (Figure 1d), which indicated a synergistic effect. The PC9/GR cells with a combination index value of 0.6 for 0.1 μM gefitinib combined with 2 μM anlotinib (Figure 1d,e) were selected for further exploration.

### Enhancement of the antiproliferative effect of gefitinib on PC9/GR cells by anlotinib

**FIGURE 2:**
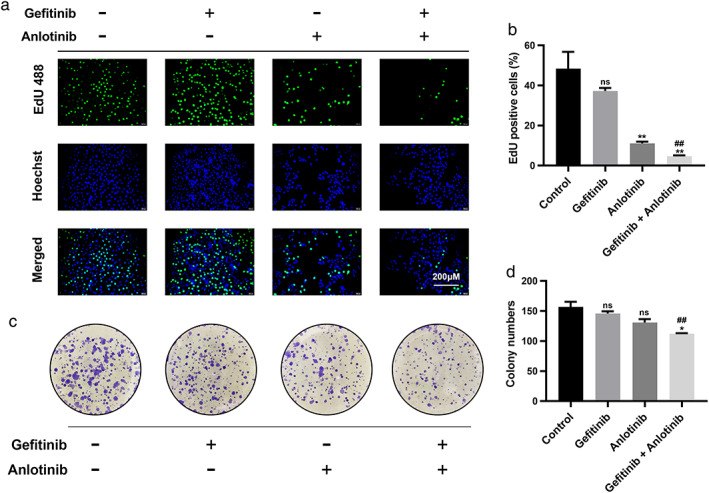
Treatment with gefitinib plus anlotinib synergistically inhibited proliferation of PC9/GR cells. (a, b) EdU proliferation assays were performed 48 h after treatment with gefitinib, anlotinib, and gefitinib plus anlotinib. (c, d) Colony‐formation assays were performed to analyze the colony‐formation efficiency of PC9/GR cells in the different treatment groups. Results are shown as mean ± SD of three independent experiments performed in triplicate. Significance levels determined using the *t* test are indicated (ns, not significant compared with the control group. *p < 0.01, **p < 0.001 compared with the control group. #*p* < 0.01, ##*p* < 0.001 compared with the gefitinib group)

To determine the antiproliferative effect of the combined treatment of anlotinib and gefitinib on PC9/GR cells, an EdU incorporation assay was performed to evaluate the activity of DNA synthesis and cell proliferation. The number of EdU‐positive cells in the combination treatment group was lower than that in the isolated 0.1 μM gefitinib treatment group (Figure 2a,b; *p* < 0.001).

A colony formation assay was then conducted to reflect the proliferative capacity of single cells. The number of colonies of PC9/GR cells were significantly reduced after combined treatments of 0.1 μM gefitinib and 2 μM anlotinib compared with isolated gefitinib treatment (Figure 2c,d; *p* < 0.001). Thus, these results demonstrated that gefitinib combined with anlotinib synergistically inhibited the proliferation of PC9/GR cells.

### Enhancement of the proapoptotic effect of gefitinib on PC9/GR cells using anlotinib

**FIGURE 3:**
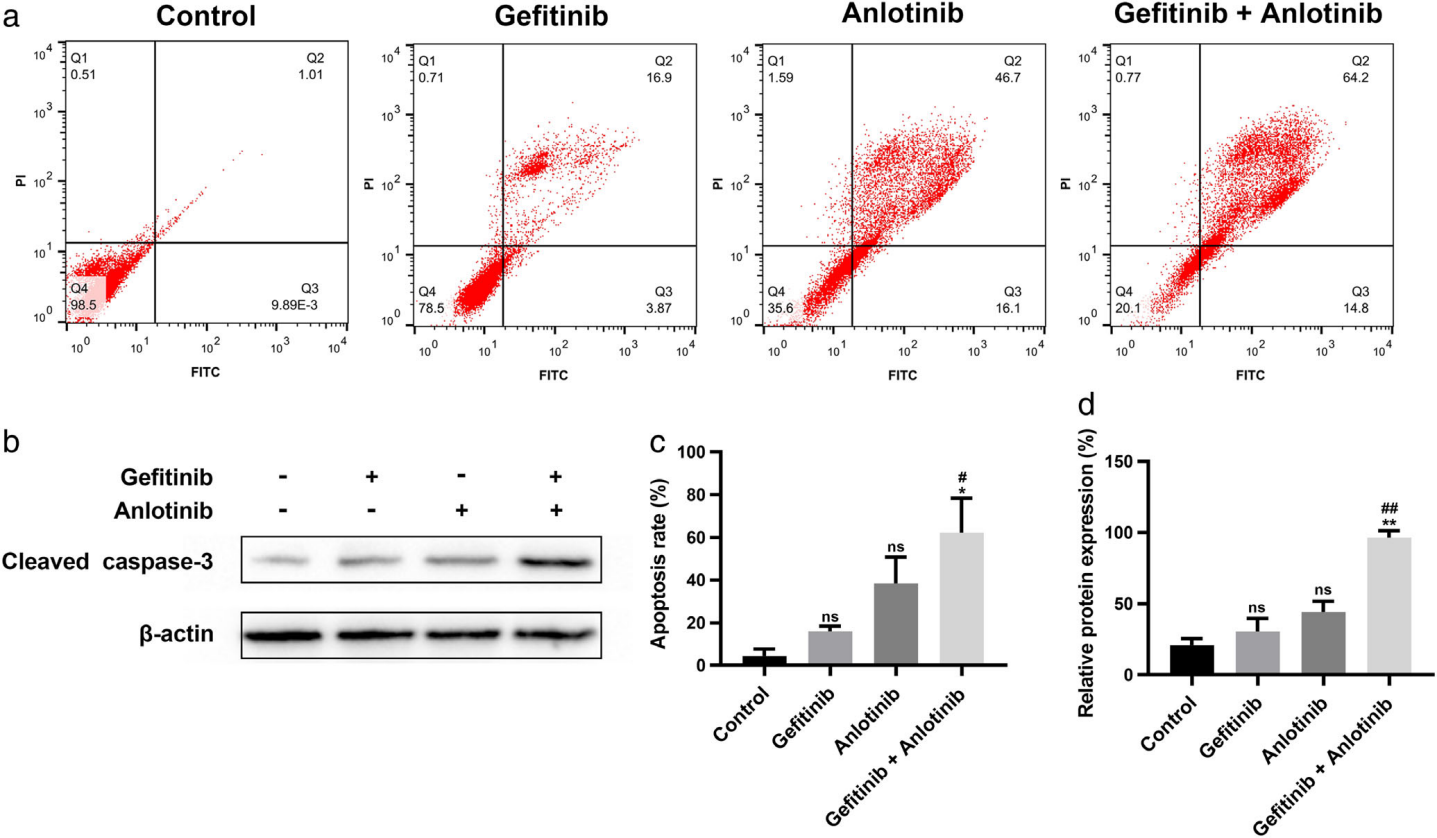
Treatment with gefitinib plus anlotinib synergistically promoted apoptosis of PC9/GR cells. (a, c) After treatment with gefitinib, anlotinib, and gefitinib plus anlotinib for 48 h, annexin V‐FITC/PI staining was used to determine the apoptosis rate of PC9/GR cells. (b, d) The apoptotic marker protein, cleaved‐caspase 3, was analyzed by western blot analysis. Significance levels determined by the *t* test are indicated (ns: not significant compared with the control group. *p < 0.05, **p < 0.01 compared with the control group. #*p* < 0.05, ##*p* < 0.01 compared with the gefitinib group)

Flow cytometric analysis was performed using Annexin V‐FITC and PI double staining to evaluate the effect of the combination on PC9/GR cells apoptosis. After treatment with 0.1 μM gefitinib alone or a combination of 0.1 μM gefitinib and 2 μM anlotinib for 48 h, we detected a significantly higher total apoptosis rate (62.3% ± 27.9%) in the PC9/GR cells of the combination group than in the gefitinib group (16.1% ± 4.1%; *p* = 0.047; Figure 3a,c). Furthermore, the protein expression of cleaved‐caspase 3 was detected as a marker for apoptosis, and the results exhibited the upregulation of protein expression level in the combination group relative to the gefitinib group (Figure 3b,d; *p* = 0.003). These results indicated that the proapoptotic ability of gefitinib was enhanced by anlotinib.

### Gefitinib and anlotinib synergistically inhibited the Akt and ERK signaling pathways

**FIGURE 4:**
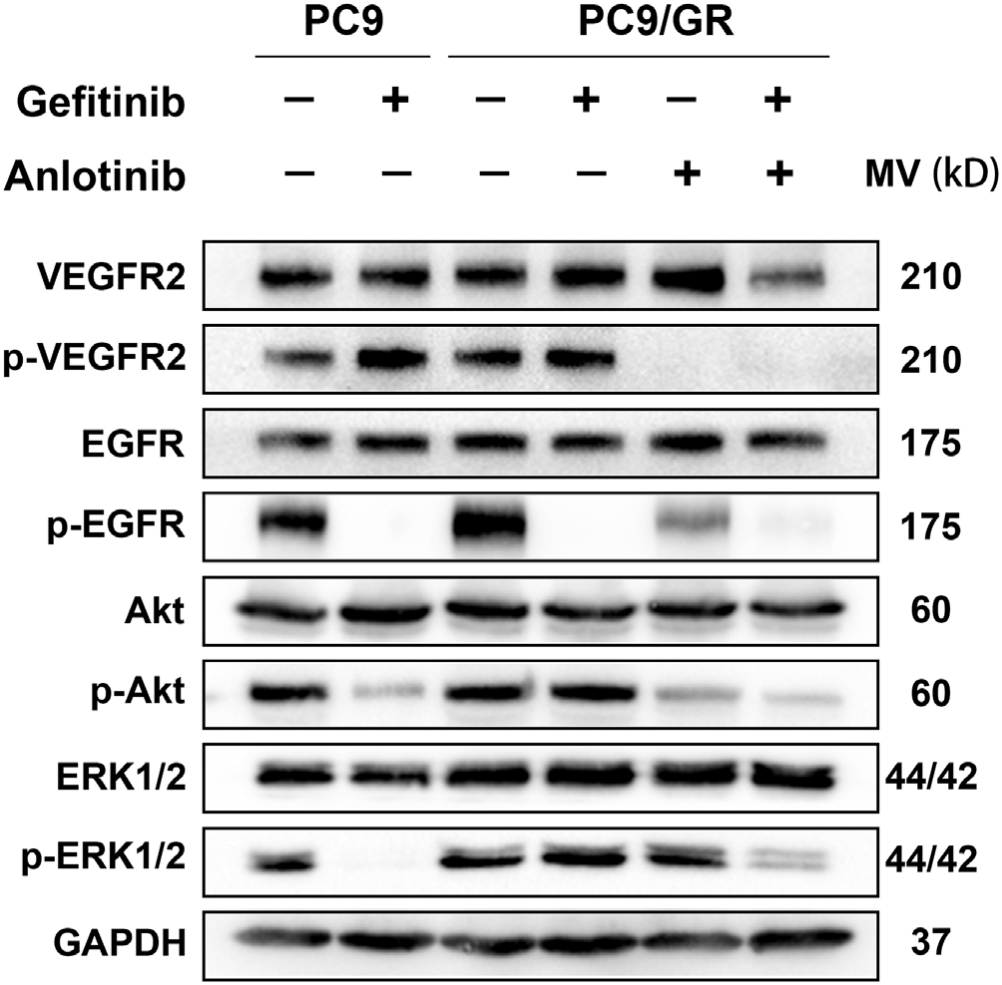
Gefitinib and anlotinib synergistically inhibited the Akt and ERK signaling pathways. After 48 h treatment with gefitinib, anlotinib, and gefitinib plus anlotinib, the key proteins involved in the EGFR and VEGFR2 downstream signaling pathways such as VEGFR2, p‐VEGFR2, EGFR, p‐EGFR, Akt, p‐Akt, ERK1/2, and p‐ERK1/2, were analyzed by Western blot analysis

The effect of gefitinib and/or anlotinib on PC9 and PC9/GR cells was further studied to investigate the mechanisms of the drug combination. Due to the tight connection between the EGFR and VEGFR signaling, protein expression levels of EGFR, VEGFR2, and their downstream signaling were determined by western blotting. After the 48‐h treatment, 0.1 μM gefitinib significantly downregulated the expression of p‐EGFR in both PC9 and PC9/GR cells (Figure 4 and Figure S1). However, decreased p‐ERK and p‐Akt expression was only observed in PC9 cells. The p‐ERK, p‐Akt, and p‐VEGFR2 expression levels in PC9/GR cells which received the combination treatment were dramatically decreased. Thus, we concluded that anlotinib reversed gefitinib resistance in PC9/GR cells through the inhibition of VEGFR2 phosphorylation and downregulation of ERK and Akt signaling (Figure S2).

### Reversal of the EGFR–TKI resistance in vivo by anlotinib

**FIGURE 5:**
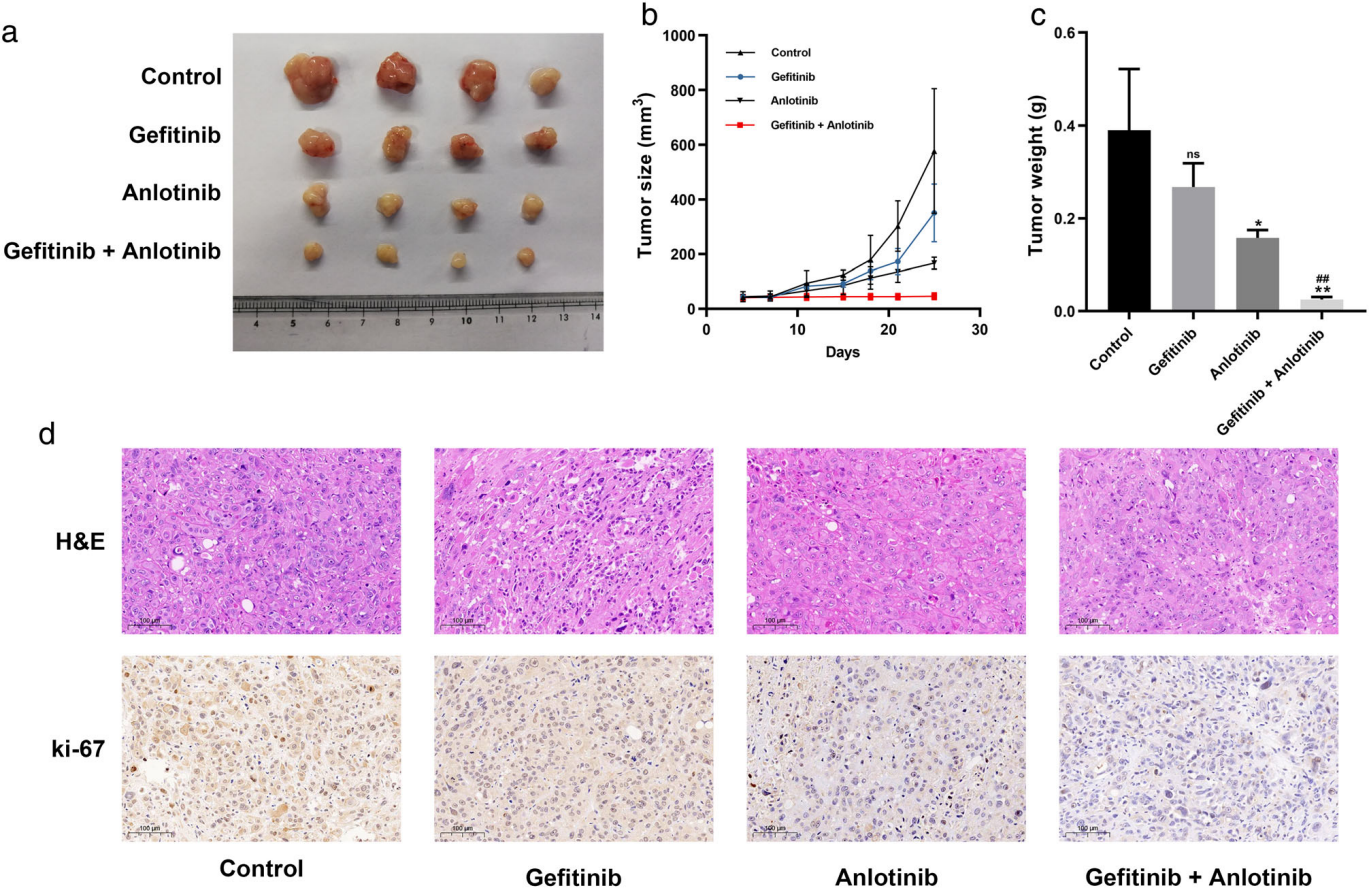
Anlotinib reversed the EGFR–TKI resistance in vivo. (a) PC9/GR cells were injected into nude mice. After four days, the mice were treated with gefitinib, anlotinib, or gefitinib plus anlotinib. (b) Tumor volume was measured every 3–4 days. (c) Tumor weight was measured after removal. (d) The tumor sections were examined using HE staining and immunohistochemical (IHC) staining with antibodies against Ki‐67. Results are shown as mean ± SD. Significance levels determined using the *t* test are indicated (ns, not significant compared with the control group. *p < 0.01, **p < 0.001 compared with the control group. #*p* < 0.01, ##*p* < 0.001 compared with the gefitinib group)

To verify the effect of anlotinib in reversing gefitinib resistance in vivo, PC9/GR cells were injected into mice to establish a tumor xenograft model. When solid tumors reached an average volume of 50 mm^3^, mice were treated with gefitinib (25 mg/kg) alone, anlotinib (6 mg/kg) alone, or gefitinib (25 mg/kg) + anlotinib (6 mg/kg). As expected, combination treatment dramatically inhibited tumor growth (Figure 5a–c) compared to gefitinib or anlotinib treatment, respectively. Ki67 IHC staining demonstrated the proliferative activity of tumors in the combination treatment group was significantly reduced compared to the single treatment group (Figure 5d). These data indicate anlotinib reversed gefitinib resistance in vivo.

### Reversal of the EGFR–TKI resistance in patients by anlotinib

The effect of anlotinib was further validated on reversal of the EGFR–TKI resistance in patients with advanced NSCLC. Of the 27 patients included between January 2018 and August 2020, one patient was lost to follow‐up, and two patients had a follow‐up shorter than 2 months. A total of 24 patients were eventually included in the final analysis. The baseline characteristics of these patients are presented in Table 1. In these patients, L858R (*n* = 13, 54.2%) was the most frequently observed *EGFR* mutation. Deletion in exon 19 was observed in eight (33.3%) patients, and three (12.5%) patients had other uncommon *EGFR* mutations (2 with G719X mutation and 1 with L861Q mutation). Most patients (*n* = 22, 91.7%) received prior first‐generation EGFR–TKI treatment such as gefitinib (*n* = 18, 75.0%), icotinib (*n* = 3, 12.5%), and erlotinib (*n* = 1, 4.2%), whereas two (8.3%) of the three patients with uncommon *EGFR* mutations received prior afatinib treatment. EGFR–TKI failure in these patients was divided into the following two modes: four patients with dramatic progression (16.7%) and 20 with gradual progression (83.3%). After imaging demonstrated disease progression, rebiopsy was performed to rule out targetable resistance mechanisms. Then, EGFR–TKI was continued at the original dose and schedule. Anlotinib was administered in combination with EGFR–TKI at a starting dose of 10 mg daily. The dosage of only one patient was decreased to 8 mg daily because of diarrhea, whereas no patients required a dosage increase.

**TABLE 1:** Baseline characteristics (*N* = 22)

Characteristic	Value
Age, median (range), year	67 (37–87)
Sex, n (%)	
Male	13 (54.2)
Female	11 (45.8)
Histology, n (%)	
Adenocarcinoma	21 (87.5)
Squamous cell carcinoma	1 (4.2)
Others	2 (8.3)
*EGFR* mutation, n (%)	
Exon 19 deletion	8 (33.3)
Exon 21 L858R mutation	13 (54.2)
Uncommon *EGFR* mutations	3 (12.5)
First prior EGFR–TKI, n (%)	
Gefitinib	18 (75.0)
Erlotinib	1 (4.2)
Icotinib	3 (12.5)
Afatinib	2 (8.3)
Prior EGFR–TKI failure mode, n (%)	
Dramatic progression	4 (16.7)
Gradual progression	20 (83.3)
Follow‐up period, median (range), month	9.1 (2.0–27.4)

*Note*: Continuous variables are given as median and the minimum/maximum range. Categorical variables are described as n (%).

Abbreviations: EGFR, epidermal growth factor receptor; TKI, tyrosine kinase inhibitor.

**FIGURE 6:**
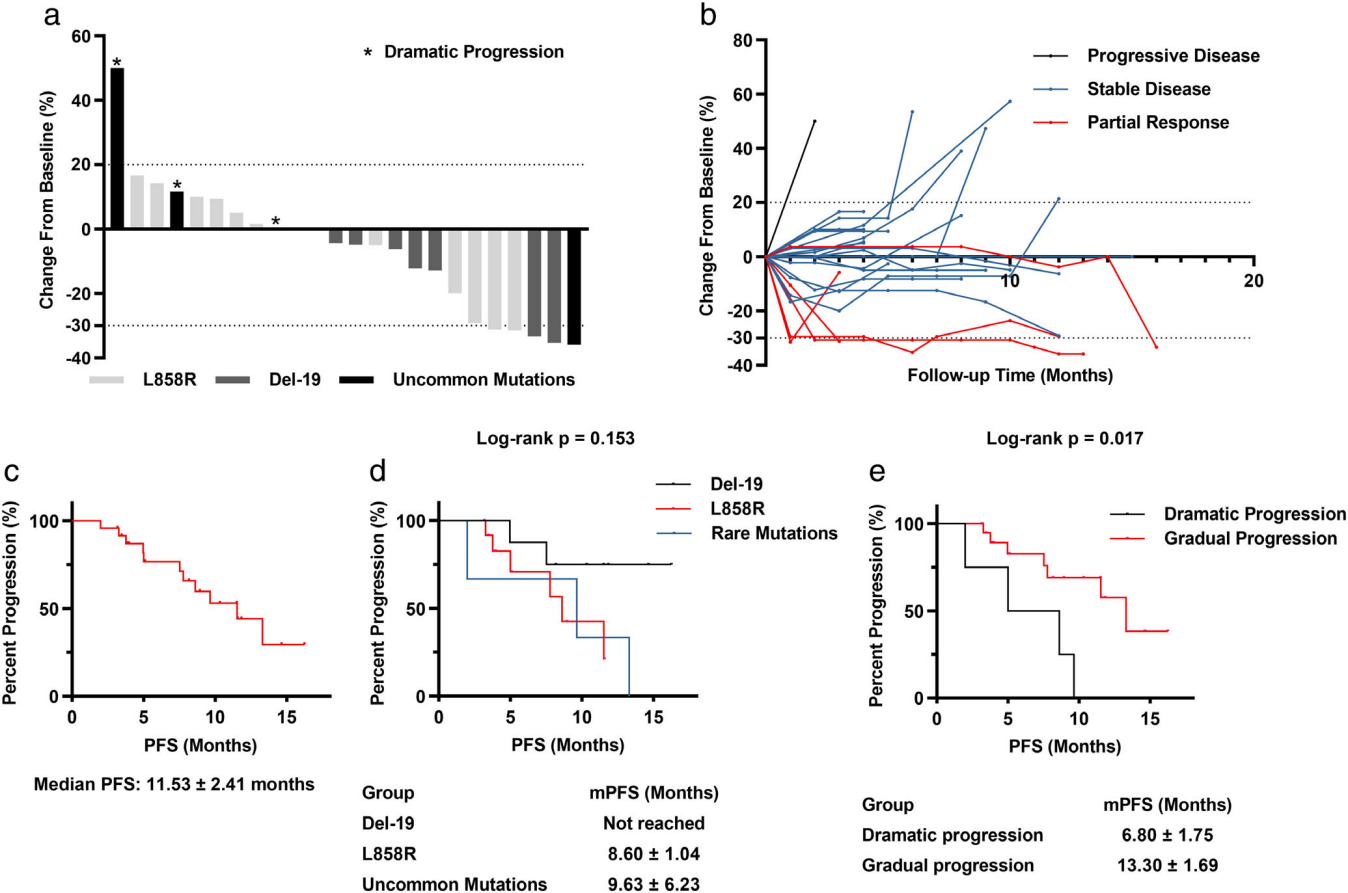
Anlotinib reversed the EGFR–TKI resistance in patients. (a) The maximal tumor shrinkage of patients receiving EGFR–TKI and anlotinib therapy. (b) The changes of tumor burden in individual patients after combination therapy. (c) Kaplan–Meier PFS curve for all patients. (d) Kaplan–Meier PFS curve stratification based on *EGFR* mutation type. (e) Kaplan–Meier PFS curve stratification based on prior EGFR–TKI failure mode

A confirmed partial response was observed in five (20.8%) patients after combination therapy, whereas 18 (75.0%) patients exhibited stable disease (Figure 6a,b). Unfortunately, one patient (4.2%) was insensitive to this combination strategy and exhibited disease progression in his first assessment, resulting in an objective response rate (ORR) of 20.8% and disease control rate (DCR) of 95.8%. The median follow‐up was 9.1 months. The median PFS was 11.53 ± 2.41 months (Figure 6c), whereas the median OS was not reached.

Subgroup analysis was performed according to the type of *EGFR* mutation. The median PFS appeared to be longer in the exon 19 deletion group (not reached) than in the L858R mutation group (8.60 ± 1.04 months) and uncommon *EGFR* mutations group (9.63 ± 6.23 months), although the difference was not statistically significant (*p* = 0.153, Figure 6d). Subgroup analysis according to the mode of EGFR–TKI failure was then performed. The median PFS in patients with gradual progression (13.30 ± 1.69 months) was much longer than that in patients with dramatic progression (6.80 ± 1.75 months, *p* = 0.017; Figure 6e).The adverse events are presented in Table 2. The most common adverse events were rash (*n* = 9, 37.5%), diarrhea (*n* = 6, 25.0%) related to EGFR–TKI and hypertension (*n* = 6, 25.0%) related to anlotinib, followed by transaminase elevation (*n* = 4, 16.7%) and fatigue (*n* = 4, 16.7%). Only one adverse event was graded as grade 3 (diarrhea, *n* = 2, 8.3%), and no adverse event was graded as grade 4 or 5. Other adverse events such as paronychia, thrombosis, bleeding, proteinuria, and leukopenia were rarely seen.

**TABLE 2:** Treatment‐related adverse events

Adverse event	Grade 1, no.	Grade 2, no.	Grade 3, no.	All grades, %
Rash	5	4	0	37.5
Diarrhea	3	1	2	25.0
Hypertension	0	6	0	25.0
Transaminase elevation	3	1	0	16.7
Fatigue	0	0	4	16.7
Bleeding	0	3	0	12.5
Proteinuria	1	1	0	8.3
Paronychia	0	1	0	4.2
Thrombosis	0	1	0	4.2
Leukopenia	0	1	0	4.2

*Note*: There were no grade 4/5 adverse events.

## DISCUSSION

Acquired resistance development is a major challenge in the EGFR–TKI treatment of NSCLC. There are different subsequent treatment options based on different mechanisms of drug resistance. Patients with *EGFR* T790M mutations often respond to third‐generation EGFR–TKIs.^6^ For patients who acquired bypass signaling pathway activation, combination therapy with bypass signaling pathway inhibitors may be beneficial.^7^, ^8^ However, there is no targeted treatment option for patients who develop resistance with an unknown mechanism but chemotherapy,^8^ and an effective therapeutic strategy must be investigated for these patients. The present study observed that gefitinib combined with anlotinib exhibited a significant antitumor effect on gefitinib‐resistant lung adenocarcinoma models in vitro and in vivo. The concurrent use of anlotinib demonstrated promising treatment effects and negligible adverse events in advanced *EGFR*‐mutant NSCLC patients after EGFR‐TKI resistance.

Anlotinib is a multitargeted small‐molecular TKI that inhibits a group of kinases such as VEGFR1/2/3, c‐Kit, and PDGFRβ.^13^ In China, this drug has been approved as third‐line treatment of locally advanced or metastatic cases of NSCLC with progression or recurrence.^13^ The ALTER 0303 clinical trial demonstrated favorable outcomes in patients with advanced NSCLC receiving anlotinib as a third‐line or further therapy.^15^ Molecular research has shown that by targeting VEGFR2, anlotinib plays antiangiogenic and antitumor roles in NSCLC.^14^ The present study observed that anlotinib induced sensitivity of the gefitinib‐resistant lung adenocarcinoma models to gefitinib by enhancing the antiproliferative and proapoptotic effects of gefitinib. Due to the close connection between EGFR and VEGFR signaling, we further performed western blotting to detect protein expression levels of EGFR, VEGFR2 and their downstream signaling. The activation of VEGFR2 and the phosphorylation of the downstream effectors, Akt and ERK, were significantly downregulated in the combination group. These results indicated that the combination treatment of anlotinib and gefitinib overcame the EGFR–TKI resistance, possibly by inhibiting the activation of VEGFR2 and the downstream Akt and ERK signaling transduction pathways.Several studies have demonstrated that EGFR–TKI combined with antiangiogenic drugs such as bevacizumab,^11^ apatinib,^12^, ^18^ and ramucirumab^19^ exhibit encouraging clinical activity in patients with NSCLC. However, there are currently few similar reports providing clinical outcomes for the combination of EGFR–TKI and anlotinib. In the present study, we retrospectively reviewed the medical records of 24 eligible patients with NSCLC who received combination therapy of EGFR–TKI and anlotinib after EGFR–TKI resistance. Combination therapy exerted favorable effects with an ORR of 20.8% and a DCR of 95.8%. Short‐term prognosis was also observed to be superior with a median PFS of 11.53 ± 2.41 months. The adverse events data suggested this regimen was well tolerated with no grade 4 or 5 adverse events reported. Thus, the combination of EGFR–TKI and anlotinib could be considered as a therapeutic salvage option for patients who develop drug resistance with an unknown mechanism.

The treatment effect of EGFR–TKI varies among patients with different *EGFR* mutations. Therefore, we performed subgroup analysis according to the type of E*GFR* mutations. The estimated median PFS was longer in the exon 19 deletion group (not reached) than in the L858R mutation group (8.60 ± 1.04 months) and uncommon *EGFR* mutations group (9.63 ± 6.23 months), although this difference was not statistically significant (*p* = 0.153). A larger sample size might demonstrate more credible results.

Yang et al. divided the diversity of EGFR–TKI failure into three modes according to specific criteria derived from clinical factors, namely dramatic progression, gradual progression, and local progression.^16^ Their study indicated that patients in the dramatic progression group demonstrated a better survival when switching to chemotherapeutic regimens, whereas continuation of EGFR–TKI achieved significantly longer OS in the patients exhibiting gradual progression. In our study, 20 patients (83.3%) experienced gradual progression, whereas the other four patients (16.7%) experienced dramatic progression. These patients received a combination therapy of EGFR–TKI and anlotinib due to refusal of treatment with chemotherapy. We further performed subgroup analysis according to EGFR–TKI failure mode and discovered that patients exhibiting gradual progression (median PFS, 13.30 ± 1.69 months) are more likely to benefit from this combination strategy than those exhibiting dramatic progression (6.80 ± 1.75 months, *p* = 0.017). The CTONG1304 clinical trial reported rechallenge with gefitinib monotherapy was effective in patients after the first‐line EGFR‐TKI treatment and second‐line chemotherapy.^20^ The median PFS was 4.4 months (95% CI: 3.2–4.8 months). Additionally, a multicenter retrospective study reported afatinib monotherapy showed promising efficacy in the rechallenge setting after acquired resistance to gefitinib or erlotinib.^21^ The median PFS was 8.0 months (95% CI: 4.9–9.5 months). Our study indicated that combination strategy of EGFR–TKI and anlotinib would be more beneficial with longer PFS (11.53 ± 2.41 months), especially in patients exhibiting gradual progression.

The ABC clinical trial of the combination of afatinib and bevacizumab after acquired resistance to EGFR–TKI in *EGFR*‐mutant NSCLC exhibited a median PFS of 6.3 months (95% CI: 3.9–8.7 months).^11^ A retrospective analysis of EGFR–TKI combined with apatinib after EGFR–TKI failure also exhibited a median PFS of 4.6 months (95% CI: 2.23–12.52 months).^12^ The present study suggested a longer median PFS of 11.53 ± 2.41 months. These results indicated concurrent use of anlotinib may be another, or even preferable, option to overcome EGFR‐TKI resistance. However, our clinical study only included patients with no targetable resistance mechanism identified by rebiopsy after acquired resistance. The ABC clinical trial and retrospective analysis of EGFR–TKI combined with apatinib included patients with *EGFR* T790M mutation. It means the differences of PFS should be interpreted with caution because T790M‐positive patients may benefit less from first‐ or second‐generation EGFR‐TKI rechallenge therapy. Further direct comparison of the effect on reversing EGFR‐TKI resistance among various antiangiogenic drugs will be of interest.

Despite the significant findings, the present study has several limitations. It was a single‐center retrospective study with a small sample size. Prospective multicenter studies with a larger sample size are required to further strengthen our findings. Moreover, we only included patients who experienced first‐ and second‐generation EGFR–TKI treatment failure. Unexpectedly, patients suffering from third‐generation EGFR–TKI treatment failure also respond to the EGFR–TKI and anlotinib combination therapy in our clinical practice. Thus, further studies on the antitumor effects of the combination of anlotinib and third‐generation EGFR–TKI would be beneficial. Furthermore, the retrospective design of the study prevented the intensive investigation of the mechanism of reversing EGFR–TKI resistance by combination therapy. We demonstrated that combination treatment of anlotinib and gefitinib inhibited the activation of VEGFR2 and the downstream Akt and ERK signaling transduction pathways in vitro. However, more in‐depth studies to clarify the molecular mechanisms are still required.

In conclusion, we demonstrated anlotinib could overcome acquired resistance to gefitinib in vitro and in vivo. The combination strategy of EGFR–TKI and anlotinib exhibited superior treatment effects and negligible adverse events in advanced *EGFR*‐mutant NSCLC patients after EGFR‐TKI resistance, especially in patients experiencing gradual progression. Our research provides a new therapeutic option to overcome EGFR‐TKI acquired resistance for this subgroup of patients.

## CONFLICT OF INTEREST

The authors declare no potential conflicts of interest.

## Supporting information

**Figure S1**. Effect of gefitinib and anlotinib on key signal transduction proteins in PC9/GR cells. PC9/GR cells were treated with gefitinib, anlotinib, or gefitinib plus anlotinib for 48 h, Western blot analysis was performed to detect the expression of key signal transduction proteins. Significance levels determined by the *t* test are indicated (ns: not significant compared with the control group. *p < 0.05, **p < 0.01 compared with the control group. #*p* < 0.05, ###*p* < 0.01 compared with the gefitinib group).

**Figure S2**. Diagram of the possible mechanism of reversing gefitinib resistance by anlotinib. Gefitinib and anlotinib synergistically promoted PC9/GR cells proliferation and inhibited PC9/GR cells apoptosis through the inhibition of EGFR phosphorylation, VEGFR2 phosphorylation and the downregulation of ERK and Akt signaling.
