# Supplementary figures and images for: Concurrent use of anlotinib overcomes acquired resistance to EGFR‐TKI in patients with advanced EGFR‐mutant non‐small cell lung cancer
Source: Thorac Cancer. 2021 Sep 12;12(19):2574–84. doi: 10.1111/1759-7714.14141 (PMC8487816; doi:10.1111/1759-7714.14141)

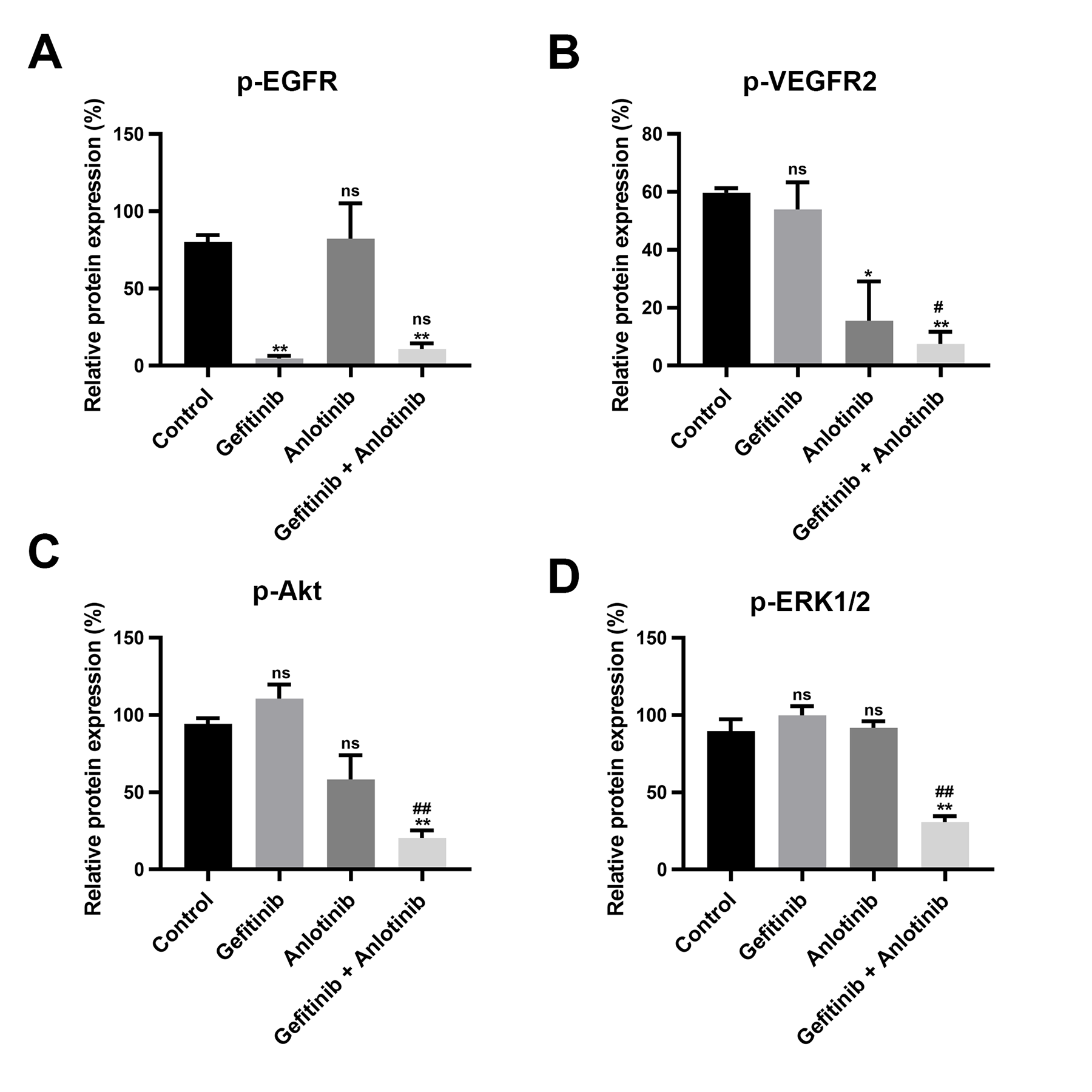

Supplement: Supplementary file 1 — Figure S1. Effect of gefitinib and anlotinib on key signal transduction proteins in PC9/GR cells. PC9/GR cells were treated with gefitinib, anlotinib, or gefitinib plus anlotinib for 48 h, Western blot analysis was performed to detect the expression of key signal transduction proteins. Significance levels determined by the t test are indicated (ns: not significant compared with the control group. *p < 0.05, **p < 0.01 compared with the control group. #p < 0.05, ###p < 0.01 compared with the gefitinib group). [file TCA-12-2574-s001.tif]

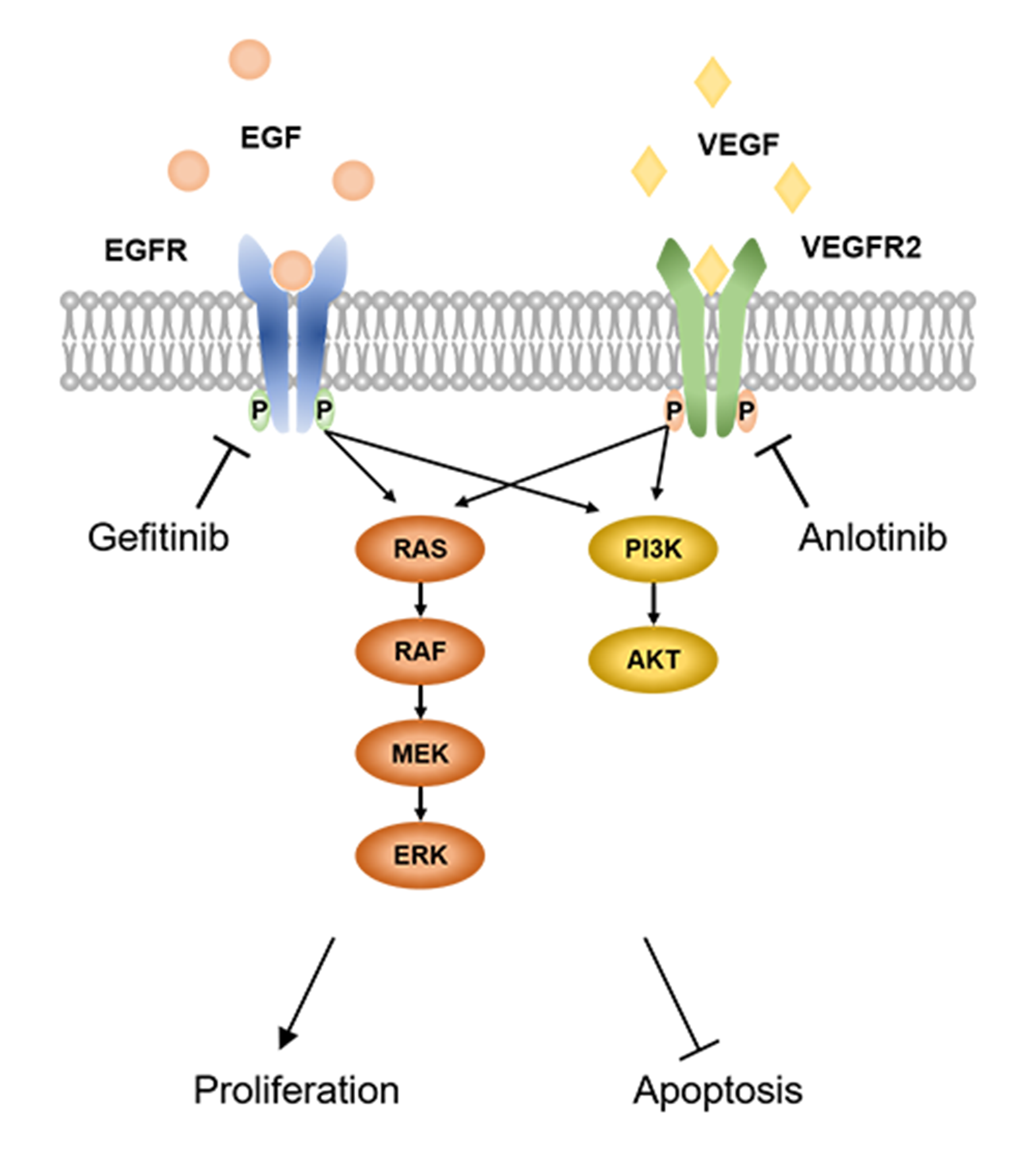

Supplement: Supplementary file 2 — Figure S2. Diagram of the possible mechanism of reversing gefitinib resistance by anlotinib. Gefitinib and anlotinib synergistically promoted PC9/GR cells proliferation and inhibited PC9/GR cells apoptosis through the inhibition of EGFR phosphorylation, VEGFR2 phosphorylation and the downregulation of ERK and Akt signaling. [file TCA-12-2574-s002.tif]
